# Prenatal Diagnosis of a Fetus with Ring Chromosomal 15 by Two- and Three-Dimensional Ultrasonography

**DOI:** 10.1155/2014/495702

**Published:** 2014-10-20

**Authors:** Ingrid Schwach Werneck Britto, Sandra Regina Silva Herbest, Giselle Darahem Tedesco, Carolina Leite Drummond, Luiz Claudio Silva Bussamra, Edward Araujo Júnior, Rodrigo Ruano, Simone Hernandez Ruano, José Mendes Aldrighi

**Affiliations:** ^1^Department of Obstetrics and Gynecology, Medical College Science of Santa Casa of São Paulo (FCMSCSP), 01221-020 São Paulo, SP, Brazil; ^2^Department of Obstetrics, Federal University of São Paulo (UNIFESP), Rua Carlos Weber 956, Apartamento 113 Visage, 05303-000 São Paulo, SP, Brazil; ^3^Department of Gynecology and Obstetrics, Baylor College of Medicine and Texas Children's Hospital, Houston, TX, USA; ^4^Department of Genetics, Federal University of São Paulo (UNIFESP), 77030 São Paulo, SP, Brazil

## Abstract

We report on a prenatal diagnosis of ring chromosome 15 in a fetus with left congenital diaphragmatic hernia (CDH) and severe intrauterine growth restriction (IUGR). A 31-year-old woman, gravida 2 para 1, was referred because of increased nuchal translucency at gestational age of 13 weeks. Comprehensive fetal ultrasound examination was performed at 19 weeks revealing an early onset IUGR, left CDH with liver herniation, and hypoplastic nasal bone. Three-dimensional ultrasound (rendering mode) showed low set ears and depressed nasal bridge. Amniocentesis was performed with a result of a 46,XX,r(15) fetus after a cytogenetic study. A 1,430 g infant (less than third percentile) was born at 36 weeks. The infant presented with respiratory failure and died at 2 h of life. Postnatal karyotype from the umbilical cord confirmed the diagnosis of 15-ring chromosome. We described the main prenatal 2D- and 3D-ultrasound findings associated with ring chromosome 15. The interest in reporting the present case is that CDH can be associated with the diagnosis of 15-ring chromosome because the critical location of the normal diaphragm development is at chromosome 15q26.1-q26.2.

## 1. Introduction

Ring chromosome 15 is a rare structural chromosomal abnormality. There are approximately 40 cases that have been reported in the literature [1]. A breakage in both arms of a chromosome, and the fusion of the points of fracture with consequent loss of the distal fragments, is the main mechanism involved in the origin of a ring chromosome [1]. This results in monosomy for the distal short arm and the distal long arm of the chromosome involved [1, 2]. In unbalanced ring chromosomes, the phenotype may vary depending in part on the loss of euchromatin distal to the breakpoints [1, 3–6]. A telomeric fusion without loss of genetic material can also be the origin of a ring chromosome. Moreover, it can occur as a supernumerary chromosome from the duplication of the chromosomal material contained within the ring [1]. The formation of rings is usually a sporadic event, but familial cases of ring chromosome have been also reported [5, 7].

The ring 15 chromosome syndrome results in a variable phenotype; however, there are some common findings. In a review of 25 cases from the literature, the main characteristics of this syndrome include intrauterine growth restriction (100%), variable mental retardation (95%), microcephaly (88%), hypertelorism (46%), and triangular facies (42%). Other frequent findings include delayed bone age (75%), brachydactyly (44%), speech delay (39%), frontal bossing (36%), anomalous ears (30%), café-au-lait spots (30%), cryptorchidism (30%), and cardiac abnormalities (30%) [8]. A genotype-phenotype correlation is determined by the extent of euchromatic loss and level of mosaicism.

Therefore, a precise genotype-phenotype correlation is usually problematic [8, 9]. Fluorescence in situ hybridization (FISH) has been used to characterize the r(15) chromosome and array comparative genomic hybridization (array-CGH) has determined the size of the 15q deletion [9, 10]. There are four cases in prenatal diagnosis reported [1, 9–12].

We reported a prenatally diagnosed fetus at 19 weeks with ring chromosome 15 and severe IUGR associated with left congenital diaphragmatic hernia (CDH), which may have particular aspects involved in the genetic disease of this anomaly.

## 2. Case Report

**Figure 1:**
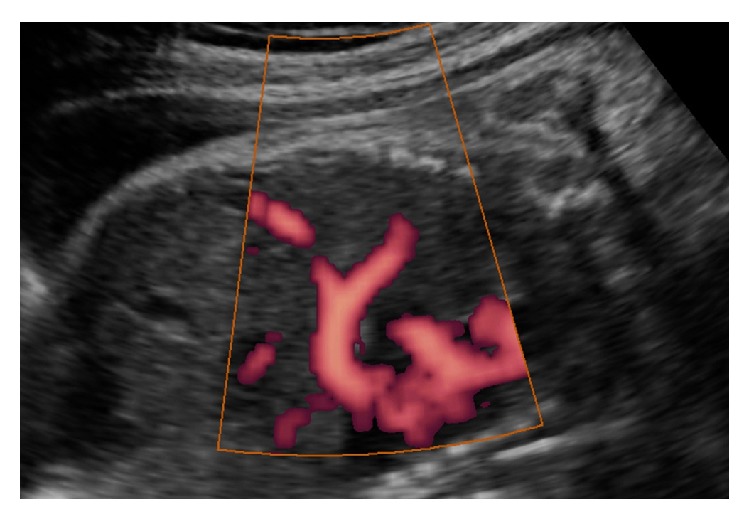
Two-dimensional ultrasound finding in a fetus with ring chromosome 15 at 19 weeks of pregnancy showing liver herniation into the thorax due to congenital diaphragmatic hernia.

A 31-year-old female, gravida 2 para 1, with a 2-year-old healthy child, was referred to our Fetal Center because of increased nuchal translucency (3 mm) at 13 weeks, measured elsewhere. Doppler study of the ductus venosus and assessment of fetal nasal bones were not evaluated at that examination. No other structural abnormalities were described at that moment. The combined test, including ultrasound and biochemistry tests, was not performed in this patient because it is not routinely offered in our country because of the elevated costs. No prenatal invasive test was indicated before the patient was observed. At referral, a fetal comprehensive ultrasound exam was performed at 19 weeks using a SonoAce X8 apparatus (Samsung, Seoul, Korea) equipped with a convex volumetric multifrequency transducer (4–7 MHz). Fetal biometry revealed a biparietal diameter (BPD) of 38 mm (17 + 6 weeks), an abdominal circumference (AC) of 110 mm (16 weeks), and a femur length (FL) of 22 mm (16 + 6 weeks), indicating fetal growth restriction. Ultrasound examination also showed a large left CDH with liver herniation into the chest and hypoplastic nasal bone (Figure 1).

The nuchal fold thickness was 2.8 mm. The amniotic fluid index and arterial Doppler were normal. Subsequent ultrasound examination also showed dolichocephaly and clubfoot. Hands were small and remained constantly flexed, but eventually opened.

**Figure 2:**
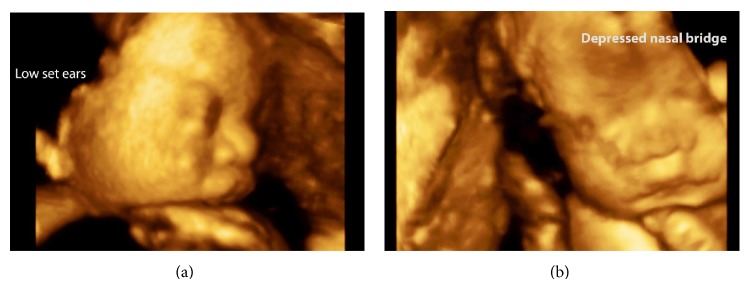
Three-dimensional ultrasound in the rendering mode findings in a fetus with ring chromosome 15 at 19 weeks of pregnancy. (a) Face fetus showing the low set ears. (b) Face fetus showing the depressed nasal bridge.

**Figure 3:**
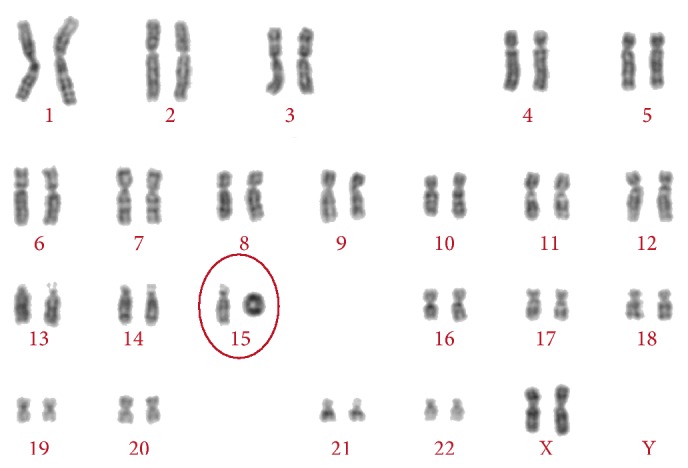
G banding of chromosomes revealed a ring chromosome 15, karyotype (46,XX,r(15)[20]).

Three-dimensional ultrasound revealed low set ears and depressed nasal bridge (Figure 2). Echocardiography was performed at 21 weeks and confirmed the cardiac axis deviation and compression of the left chambers due to congenital diaphragmatic hernia. No structural cardiac anomalies were observed except the disproportion of the heart chambers by compression of the herniated organs into the chest. Cardiac function was preserved. For legal reasons, termination of the pregnancy was not an option. Parents elected to proceed with amniocentesis for fetal karyotype at 22 weeks for prenatal genetic counseling. Fetal karyotype (G-banding or Giemsa banding) revealed a ring chromosome 15 (46,XX,r(15)[20]) (Figure 3). However, we were not able to perform an array comparative genomic hybridization to determine the extension of the chromosomal abnormality. After genetic counseling, both parent's karyotyping examinations were performed and the results were normal.

Because there was a chromosomal abnormality but it was not possible to establish the prognosis, fetal surveillance was performed by fetal Doppler studies every 2 weeks because of the diagnosis of IUGR. The last ultrasound exam was performed at 36-week-gestation age, and fetal biometry revealed a BPD of 76 mm (30 + 3 weeks), an AC of 248 mm (29 weeks), and a FL of 54 mm (29 + 6 weeks), indicating severe IUGR.

A 1,430 g newborn (less than third percentile) was delivered by cesarean section at 36 weeks because of abnormal Doppler studies, following patient request. Apgar scores were 3, 6, and 7 at first, third, and fifth minutes of life. The newborn developed severe respiratory failure, leading to death in 2 h. All dysmorphic findings were observed after birth. Autopsy was not performed because the parents denied it. In addition, the parents denied any further genetic study on the fetus. The karyotype collected from the umbilical cord confirmed the diagnosis of 15-ring chromosome.

## 3. Discussion

Ring chromosome 15 is a rare structural chromosomal abnormality that results from the loss of distal ends of both the p arm and q arm with union of both ends. There are four cases in prenatal diagnosis reported [1, 9, 11, 12].

Liu et al. [11] reported a 27-year-old woman, gravida 2, para 1, with a fetus at 20-week gestation age with increased nuchal fold up to 8 mm. Fetal ultrasound exam showed IUGR. Karyotype was 46,XY, r(15) (p13q26), and FISH demonstrated the loss of the telomeric region of 15 q arm. The pregnancy was terminated at 23-week gestation.

Glass et al. [9] described another 16-week fetus with thickened nuchal fold, single umbilical artery, and IUGR. Amniocentesis was performed and showed mosaicism and FISH analysis showed loss of the subtelomeric region from the r(15) chromosome. Caesarian section was performed at 32 weeks due to oligohydramnios and IUGR. The child was born with growth restriction (less than third percentile), incomplete atrioventricular canal, atrial septal defect, mild left pulmonary artery stenosis, and mild hypoplastic mitral valve. Other findings were prominent occiput, rotated ears, short neck, small hands, brachydactyly, clinodactyly, and conductive hearing loss. At 6 years old, the child was measuring 9.98 kg, 56 cm, and had speech delays.

Hatem et al. [12] described a woman 27 years-old, gravida 1, para 0, with a fetus at 18-week gestation with IUGR, oligohydramnios, CDH with liver herniation, and polycystic kidneys. Karyotype was 46,XX,r(15) and FISH revealed loss of 15q26.1-qter region. The pregnancy was terminated at 22-week gestation.

Manolakos et al. [1] reported a fetus with normal nuchal translucency, with chorionic villus sampling, showing a ring chromosome 15 in two different cultures and karyotype 46,XX,r(15)[30]. At 15 weeks, an amniocentesis was performed and showed ring chromosome 15 in mosaicism with monosomy 15 cell line, and karyotype was 46,XX,r(15)(p11.1q26.3)[21]/45,XX,-15[9]. The parents decided to terminate the pregnancy at 18 weeks. Necropsy showed a fetus with dolichocephaly, triangular face, Micrognathia, carpal flexion, clinodactyly, clubfoot, and diaphragmatic hernia.

According to previous reports in the literature, infants with ring chromosome 15 are compatible with life but usually have severe growth restriction during childhood. Although ring chromosome 15 is related to variable phenotypes, most recurrent findings are severe growth deficiency, mental retardation, and dysmorphic features [12]. Severe growth deficiency is a common findings in the ring chromosome 15 because of loss of the insulin-like growth factor I receptor gene located in 15q26.3 [1, 9, 10, 12].

IUGR is a common finding in fetuses with abnormal karyotype [11]. CDH is an uncommon finding in ring chromosome 15 [6] and it occurs because of the critical location of the normal diaphragm development at chromosome 15q24–26. Recent studies have shown that a critical region implicated in CDH is localized in 15q26.1-q26.2 [13–18]. Here we reported a fetus with both findings, severe IUGR and left CDH, which may be explained by possible deletions in regions of chromosome 15 that may be crucial to the development of the diaphragm.

In the present case, three-dimensional ultrasound was helpful to provide more information on the fetal face and limbs. Three-dimensional ultrasound in the rendering mode was important in the assessment of facial deformations in the present fetus (low-set ears and depressed nasal bridge).

Most of the cases of ring chromosome 15 are diagnosed postnatal. Except for the diagnosis of IUGR, there are no specific ultrasound findings described in the literature that are related to this syndrome [1]. However, we reported a new case of prenatal diagnosis of ring chromosome 15 associated with severe phenotypes including IUGR, left CDH, dolichocephaly, depressed nasal bridge, and clubfoot, which may be associated with poor prognosis.

We reported a new case of prenatal diagnosis of ring chromosome 15 associated with severe phenotypes.
